# Does Aging Affect PolyJet™ 3D-Printed Teeth for Endodontics? A Micro-CT Evaluation

**DOI:** 10.3390/jfb17050224

**Published:** 2026-05-02

**Authors:** Cláudia Barbosa, Tiago Reis, José B. Reis, Margarida Franco, Catarina Batista, Rui B. Ruben, Benjamín Martín-Biedma, José Martín-Cruces

**Affiliations:** 1 Endodontics and Restorative Dentistry Unit, School of Medicine and Dentistry, University of Santiago de Compostela, 15701 Santiago de Compostela, Spain; cbarbosa@ufp.edu.pt (C.B.); pepe3214@gmail.com (J.M.-C.); 2 FP-I3ID, FP-BHS, Faculty of Health Sciences, University Fernando Pessoa, 4200-150 Porto, Portugal; 3 RISE-Health, Faculty of Health Sciences, University Fernando Pessoa, Fernando Pessoa Teaching and Culture Foundation, 4200-150 Porto, Portugal; 4 CDRSP, Polytechnic University of Leiria, 2430-028 Marinha Grande, Portugal; margarida.franco@ipleiria.pt (M.F.); catarina.batista@ipleiria.pt (C.B.); rui.ruben@ipleiria.pt (R.B.R.); 5 Faculty of Engineering, University of Porto, 4200-465 Porto, Portugal; jose.b.reis05@gmail.com; 6 Oral Sciences Research Group, Endodontics and Restorative Dentistry Unit, School of Medicine and Dentistry, University of Santiago de Compostela, Health Research Institute of Santiago de Compostela (IDIS), 15706 Santiago de Compostela, Spain; benjamin.martin@usc.es

**Keywords:** PolyJet™, 3D printing, aging, 3D printed teeth, endodontics

## Abstract

This study aimed to evaluate the aging effect (6 and 12 months), relative to baseline (0 months), on the dimensional accuracy, morphological stability, and shaping behavior of PolyJet™ 3D-printed teeth (3DPT) produced in two printing orientations (*X* and *Y* axes). Specimens (XA0, XA6, XA12, YA0, YA6, YA12) were analyzed using microcomputed tomography before and after root canal preparation with the ProTaper Gold^®^ system. Preoperative analysis included canal volume, centroid, total tooth volume, and total tooth area. Aging-related changes were observed, with significant differences between XA0 and XA12 (*p* < 0.05), whereas no differences were detected among *Y*-axis groups (*p* > 0.05). These findings indicate that *X*-axis specimens are not comparable over time, while *Y*-axis specimens maintain baseline consistency. Postoperative evaluation revealed significant differences across aging conditions for most endodontic preparation parameters. Within the limitations of this study, aging had a limited effect on dimensional accuracy but influenced the shaping behavior of 3DPT. Based on these findings, future studies using PolyJet™ 3DPT should report the printing batch and the storage time between fabrication and experimental use, as these factors may influence the comparability and reliability of the results.

## 1. Introduction

Three-dimensional (3D) printing emerged from the need to produce 3D specimens not only for physical visualization of designs and concepts, but also as functional end-use parts with the performance and mechanical properties required for practical application [1]. PolyJet™ 3D printing technology (Stratasys Ltd., Eden Prairie, MN, USA) is based on the layer-by-layer deposition of liquid photopolymer resins that are rapidly cured using ultraviolet light. During fabrication, multiple print heads move across the build platform, forming successive layers as the platform gradually lowers in the vertical direction. This process enables the production of highly detailed structures, with very fine spatial resolution in both horizontal and vertical dimensions [2,3,4]. When complex geometries such as internal cavities or overhanging features are required, a temporary support material is printed simultaneously to preserve shape accuracy [3]. Owing to its high precision and geometric flexibility, PolyJet™ has become particularly valuable for the fabrication of medical prototypes and anatomical models [2,5]. Within this context, evaluating the mechanical properties of the 3D-printed specimens is essential [4].

Several key factors influence the mechanical properties of specimens produced by 3D printing, including building parameters, building orientation, post-curing processes, and material aging [1,6,7]. Understanding how these factors interact and produce their effects is therefore essential for optimizing the performance and reliability of printed specimens. In this way, building parameters should therefore be optimized to ensure the production of accurate specimens with consistent and homogeneous material properties [6].

Another reason 3D printing is a viable alternative to conventional manufacturing processes is its suitability for small-batch and fully customized production, a core principle of flexible manufacturing that enables reduced production time and costs while adapting efficiently to rapidly changing demand [2]. Within this framework, it is essential to integrate sustainability principles—Reduce, Reuse, and Recycle—when evaluating material sustainability and the potential reuse of 3D-printed specimens [5]. While PolyJet™ printed specimens are visually precise, their long-term mechanical performance is a critical concern given their high production costs and environmental impact [8], as PolyJet™ materials possess a limited serviceable lifespan defined by their expiry date. Their use prior to expiration is required; however, because replacing material cartridges in the printer entails substantial cost, there is a strong operational incentive to fabricate large batches of specimens in a single production cycle to reduce waste and avoid additional financial burden [9]. For large-scale production and practical application, it is essential that their mechanical properties remain consistent and accurately reproduced, both immediately after fabrication and throughout their service life. In this context, dimensional accuracy refers to the degree to which the printed model reproduces the original geometry at a given time point, whereas morphological stability describes the ability of that geometry to remain unchanged over time, particularly under aging conditions—an aspect that is critical in demanding applications such as medical training and research [8]. Aging encompasses changes in material behavior resulting from environmental exposure and internal chemical processes [1,7]. Aging is a critical factor influencing mechanical (e.g., strength, toughness, hardness), physical (e.g., density), and chemical properties of polymers over time. This process may result from environmental exposure, oxidative reactions, and chemical changes occurring during curing and cooling, often acting simultaneously [10]. Consequently, it is essential to consider the combined effects of aging, degradation conditions, and manufacturing parameters when evaluating the mechanical performance of these materials [7]. It is therefore essential to evaluate how the properties of 3D-printed specimens evolve over time and how these changes influence their long-term durability and functional performance [2]. Most materials undergo natural aging at room temperature; however, aging can be accelerated under controlled moisture or temperature conditions. Under ambient conditions, polymers degrade more rapidly than other engineering materials resulting in a progressive decline in their mechanical and surface properties over time [2,11,12].

3D-printed teeth (3DPT), generated from micro-computed tomography (micro-CT) scans of natural teeth (NT), have emerged as a reliable alternative for both research and educational applications. These replicas facilitate the production of standardized and highly reproducible specimens and support the creation of well-balanced experimental groups for research [3]. The quality and functional behavior of PolyJet™ 3DPT is strongly influenced by manufacturing parameters [8]. In our previous work [6], we showed that both the orientation of 3DPT and, in particular, their position on the build platform significantly affected printing accuracy and reproducibility. Furthermore, these process variables may govern not only the initial properties of PolyJet™ 3DPT but also their performance following aging.

A literature screening (January 2026) in PubMed and Web of Science indicates that, while research on 3D printing and aging is well represented, studies specifically addressing PolyJet™ technology are limited. When further focusing on endodontic applications, no studies were identified, highlighting a clear gap in the literature. Despite the extensive body of research on aging effects across materials, this lack of evidence underscores the need for focused investigation of PolyJet™ materials within an endodontic context. While our previous work [6] established that printing axis and position affect the accuracy of PolyJet™ 3DPT, the present study is the first to assess the impact of aging on these 3DPT. This is critical for endodontic research, where standardization is essential, as time-dependent alterations in 3DPT may compromise the reliability and reproducibility of experimental results.

The primary aim of this study was to evaluate the effect of aging at 6 and 12 months on the anatomical integrity and morphological stability of PolyJet™ 3DPT manufactured in accordance with our previous work [6] in two printing orientations (*X* and *Y* axes) while maintaining the anatomical variation parallel to the build platform, using 3DPT assessed immediately after printing (0 months) as the baseline reference. Specifically, this investigation sought to determine whether aging influences external geometry, internal root canal morphology, and anatomical repeatability of 3DPT over time, thereby assessing their long-term suitability for endodontic research applications. The corresponding null hypothesis was that no significant anatomical or morphological differences would be observed across aging periods or printing orientations. The secondary aim was to analyze the shaping behavior and material response of aged 3DPT during endodontic preparation using the ProTaper Gold^®^ (Dentsply-Sirona, Fair Lawn, NJ, USA) (PTG) system, based on micro-CT evaluation of canal shaping outcomes. The associated null hypothesis was that aging would not affect the shaping behavior of 3DPT during endodontic preparation.

## 2. Materials and Methods

The experimental protocol was reviewed and authorized by the Ethics Committee of Fernando Pessoa University (approval code FCS/PI 636/24). Prior to specimen preparation, the required sample size was determined using G*Power software (version 3.1.9.7 for Windows; Heinrich Heine University, Düsseldorf, Germany), drawing on effect estimates reported in earlier research on shaping performance [13]. The analysis was carried out within the t-test framework, considering an effect magnitude of 1.79, a type I error probability of 0.05, and a statistical power of 95%. This calculation indicated that at least 16 specimens were necessary, evenly distributed between groups. To compensate for possible losses throughout the experimental workflow, each group ultimately included 10 samples.

**Figure 1:**
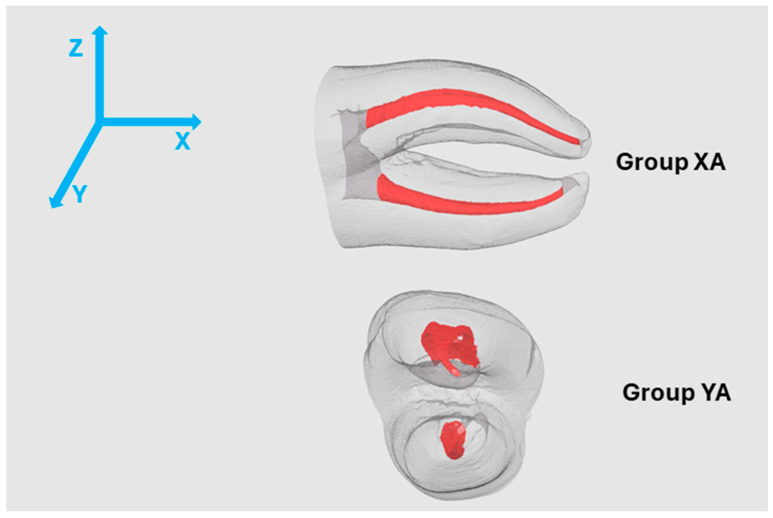
Orientation of the natural tooth on the build platform (frontal view) with long-axis parallel to the X, Y and the isthmus parallel to the build platform (adapted from Barbosa et al. (2025) [6]).

The 3DPT used in the present study were produced in the same manufacturing batch as those reported in our previous study [6], in which printing orientation and build position were evaluated. Based on those findings, the optimal build position was selected, and specimens printed along the *X* and *Y* axes and A position, with the isthmus parallel to the build platform were retained for assessment for the present study (Figure 1). The 3DPT were analyzed immediately after printing as the baseline condition (0 months), and independent specimens from the same printing batch were evaluated after 6 and 12 months of aging to assess time-dependent changes, resulting in six experimental groups: XA0, XA6, XA12, YA0, YA6, and YA12.

The aging groups 3DPT were stored for 6 and 12 months at room temperature in a university laboratory under uncontrolled environmental conditions, with natural fluctuations in temperature and relative humidity. This approach was intentionally adopted to replicate typical storage conditions encountered in academic research settings and to evaluate material behavior under everyday indoor conditions [1,10]. The specimens were stored in a closed opaque plastic container to minimize uncontrolled light exposure, without direct sunlight, while still being subjected to natural daylight and artificial indoor lighting. Throughout the storage period, temperature ranged between 5 and 30 °C in accordance with seasonal variations.

### 2.1. 3DPT Preparation

All 3DPT groups underwent the root canal preparation protocol at their corresponding evaluation time points, including the baseline condition (0 months) and at 6 and at 12 months of aging.

The support material was removed according to the protocol described by Reis et al. [14]. This process resulted in a total of 20 3DPT for evaluation at each observation time point, prior to the baseline micro-CT evaluation.

All specimens were imaged using the same micro-CT system (Skyscan 1174; Bruker, Kontich, Belgium). Acquisition parameters were set at 50 kV and 800 mA, with a rotational step of 0.9° over a total of 180°. Images were obtained at a voxel size of 19.60 μm and an exposure time of 5000 ms. The resulting projections were reconstructed with NRecon software (v1.7.46; Bruker), incorporating corrections for ring artifacts (5), smoothing (3), and beam hardening (50%).

### 2.2. Root Canal Preparation

All endodontic procedures were performed by a single experienced operator with over 20 years of clinical practice and prior experience with the PTG system. The working length was determined by subtracting 1 mm from the point at which a #10 K-file (Dentsply Sirona, Charlotte, NC, USA) was visible at the apical foramen. Specimens were stabilized using the ProTrain^®^ system (Simit Dental Srl, Mantua, Italy). Canal preparation was carried out with an X-Smart^®^ Plus motor (Dentsply Sirona, Charlotte, NC, USA) in continuous clockwise rotation, following the manufacturer’s instructions.

### 2.3. Preparation of 3DPT with PTG

A glide path was initially established using the ProGlider^®^ instrument (Dentsply Sirona, Charlotte, NC, USA) up to the predetermined working length. Canal preparation was subsequently performed with the PTG sequence (SX, S1, S2, F1, and F2). Apical patency was verified after each instrument using a #10 K-file, and each set of instruments was used for a single specimen before disposal.

Irrigation was carried out between each instrument change with 5 mL of distilled water delivered via an Irriflex^®^ needle (Produits Dentaires SA, Vevey, Switzerland), followed by ultrasonic activation using the EndoActivator^®^ system (Dentsply Sirona, Charlotte, NC, USA) with a small tip at high frequency for 30 s. Upon completion of instrumentation, two additional activated irrigation cycles of 5 mL each were performed, resulting in a total irrigant volume of 40 mL per canal. The canals were then dried with sterile paper points (Dentsply Sirona, Charlotte, NC, USA). Post-instrumentation micro-CT scans were acquired using the same parameters applied in the pre-instrumentation analysis.

### 2.4. Micro-CT Evaluation

All micro-CT analyses were carried out by a single blinded examiner. Pre- and post-instrumentation datasets were aligned using the three-dimensional registration tool available in DataViewer software (v1.5.6.2; Bruker). Quantitative measurements were then performed with CTAn software (v1.20.3.0; Bruker). The region of interest was defined from the furcation level to the apical end, following previously established protocol [14].

Centroid deviation was calculated by subtracting pre-preparation (pre) coordinates from post-preparation (post) values. Canal transportation was determined as the three-dimensional vector distance between centroids according to the formula:

$$\sqrt{{\left(\mathrm{Xpost}-\mathrm{Xpre}\right)}^{2}+{\left(\mathrm{Ypost}-\mathrm{Ypre}\right)}^{2}+{\left(\mathrm{Zpost}-\mathrm{Zpre}\right)}^{2}}$$

The proportion of untouched canal surface was computed as the ratio of static surface voxels to total surface voxels. This was obtained by subtracting prepared canal voxel counts from those of unprepared canals and converting the resulting difference into a percentage. Total tooth volume and total tooth area were determined after digitally filling the canal spaces using CTAn software.

### 2.5. Statistical Analysis

Statistical analyses were performed using SPSS software (version 30.0; IBM Corp., Armonk, NY, USA). Data normality was verified with the Shapiro–Wilk test. As all variables exhibited normal distributions, intergroup comparisons among more than two groups were conducted using one-way analysis of variance (ANOVA). When significant differences were identified, Tukey’s post hoc test was applied to determine which specific group pairs differed while controlling multiple comparisons. Independent-samples t tests were used for comparisons between two unrelated groups.

Results are presented as mean ± standard deviation, together with median and range values. Statistical significance was established at *p* < 0.05. Boxplots were used to illustrate data dispersion across the 3DPT groups and in comparison, with NT, whose reference values were obtained in our previous work [6]. For each printed group, absolute and relative biases were determined by comparing 3DPT measurements with the corresponding values from the natural tooth (NT). Method agreement was further evaluated using Bland–Altman analysis, including the calculation of mean bias and 95% limits of agreement (LoA). Relative variability was assessed by computing the coefficient of variation (CV), defined as (SD/mean × 100).

## 3. Results

**Figure 2:**
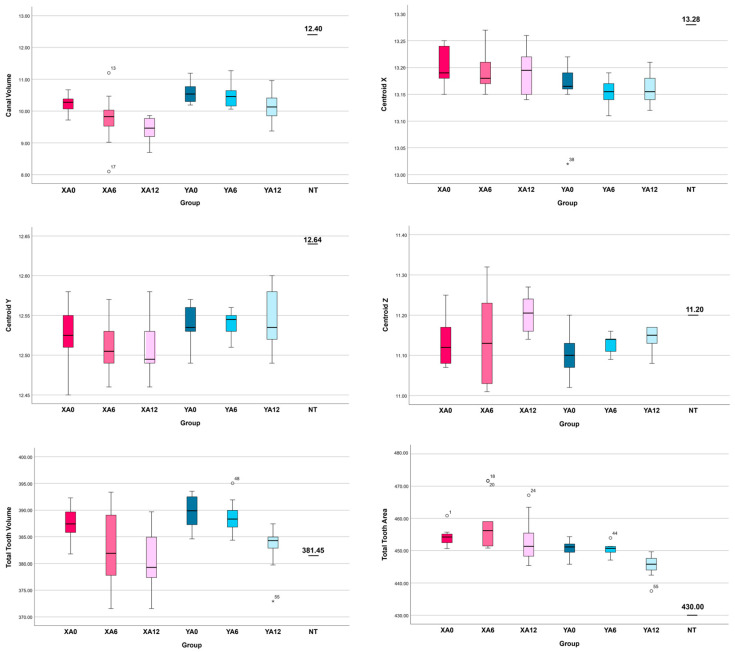
Data distribution and variability among the 3D-printed teeth groups (XA0, XA6, XA12, YA0, YA6, YA12) and relative to the natural tooth (NT).

**Figure 3:**
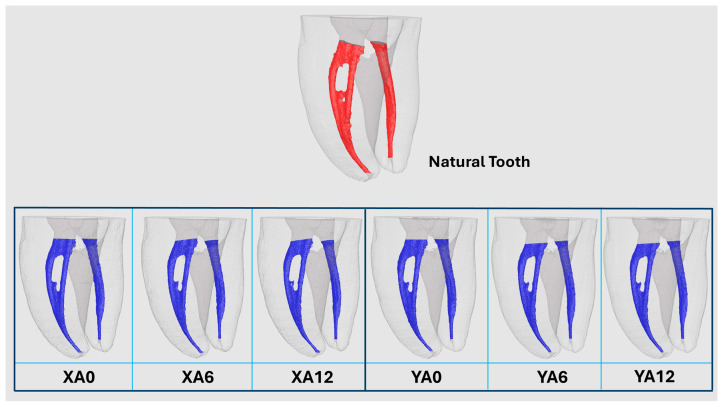
Representative 3D reconstruction of micro-computed tomography scans of samples from each 3D-printed teeth group.

The quantitative results for canal volume, centroid X, Y, and Z before endodontic preparation, total tooth volume, and total tooth area for both the NT and 3DPT groups are summarized in Table 1. The distribution patterns and variability within each group are depicted through boxplot representations (Figure 2). Additionally, Figure 3 presents representative three-dimensional micro-CT reconstructions illustrating samples from the different 3DPT groups.

**Figure 4:**
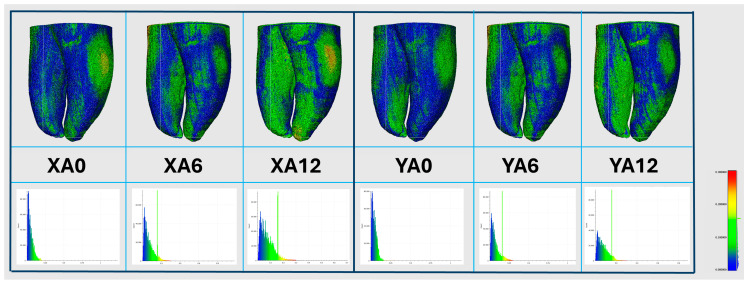
Representative micro-computed tomography reconstructions for each printing group, showing the 3D deviation map (**top panel**) and the corresponding histogram of deviation values (**bottom panel**).

Table 2 reports the absolute and relative biases between NT and the corresponding 3DPT groups, together with the 95% LoA. Canal volume showed negative biases in all groups. Centroid X and centroid Y also presented negative biases across all groups, while centroid Z showed negative biases in most groups, with a slight positive bias observed in the XA12 group. Total tooth volume and total tooth area generally exhibited positive biases except for the group XA12 that presented a negative bias for total tooth volume. With respect to aging, the magnitude of the negative bias for canal volume increased from A0 to A12 in the *X*-axis groups, while the *Y*-axis groups showed smaller variations across the aging conditions. For total tooth volume and total tooth area, the magnitude of the positive bias decreased with aging in both *X*- and *Y*-axis orientations. Figure 4 illustrates the corresponding 3D deviation map, generated in comparison with the natural tooth, and the histogram of deviation values (bottom panel) across the different groups.

The CV was calculated to assess the relative variability of total tooth volume and total tooth area measurements across the different groups (Table 3). Overall, low CV values were observed for both parameters, indicating limited variability among the printed models. The XA0 and YA0 groups showed the lowest variability for total tooth volume, while slightly higher CV values were observed in the aged groups, particularly in XA6 and XA12. A similar pattern was observed for total tooth surface area, with the lowest variability detected in the YA groups and higher dispersion in the aged XA groups.

Intergroup comparisons required the absence of statistically significant differences in the four baseline variables—canal volume and centroid X, Y, Z—to ensure that all groups started from comparable anatomical conditions. This condition was essential to attribute any subsequent changes in canal volume, transportation, and untouched areas exclusively to the endodontic preparation. In the *X*-axis groups, no statistically significant differences were found between XA0 and XA6 (*p* > 0.05) or between XA6 and XA12 (*p* > 0.05). However, XA12 differed significantly from XA0 in canal volume (*p* < 0.05), which precluded a direct comparison between these two groups, although both remained comparable with XA6. In contrast, no statistically significant differences (*p* < 0.05) were observed among YA0, YA6, and YA12, indicating that all *Y*-axis groups were fully comparable and suitable for subsequent analyses.

**Figure 5:**
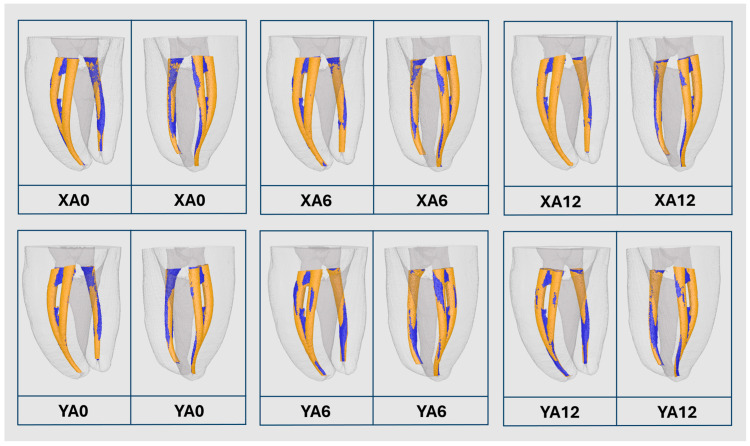
Representative 3D reconstruction of micro-CT scans before (blue) and after (orange) endodontic preparation of groups XA0, XA6, XA12, and YA0, YA6, YA12, from different views.

The comparative analysis before and after canal preparation with the PTG system is presented in Table 4. In the *X*-axis groups, statistically significant differences were identified between XA0 and XA6 (*p* < 0.05), as well as between XA6 and XA12 (*p* < 0.05), for all evaluated variables except canal transportation (*p* > 0.05). In the *Y*-axis groups, significant differences were observed for most parameters. The percentage increase in canal volume differed significantly across all groups (*p* < 0.05). For the percentage of unprepared area, differences were found between YA0 and YA6 (*p* < 0.05) and between YA6 and YA12 (*p* < 0.05), whereas no significant difference was observed between YA0 and YA12 (*p* > 0.05). Regarding canal transportation, significant differences were detected between YA0 and both aged groups (*p* < 0.05), while no differences were observed between YA6 and YA12 (*p* > 0.05). Similarly, centroid deviations along the *X* and *Y* axes differed significantly between YA0 and both YA6 and YA12 (*p* < 0.05), with no differences between the YA6 and YA12 (*p* > 0.05). For the *Z*-axis, a significant difference was observed only between YA0 and YA12 (*p* < 0.05) (Figure 5).

## 4. Discussion

The primary aim of the present study was to evaluate the effect of aging at 6 and 12 months on the anatomical integrity and morphological stability of PolyJet™ 3DPT, using specimens assessed immediately after printing (0 months) as the baseline reference. Specifically, the study investigated whether aging influences external geometry, internal root canal morphology, and the anatomical repeatability of the manufactured 3DPT. The corresponding null hypothesis assumed that no significant anatomical or morphological differences would occur across aging periods or printing orientations. The results demonstrated that aging was associated with measurable changes in certain anatomical parameters of the 3DPT. Although the magnitude of these variations remained small, statistically significant differences were detected between XA0 and XA12, which precluded direct comparison between these two groups. Consequently, the null hypothesis was only partially supported. Aging-related changes were mainly detected in canal volume and centroid deviation, suggesting that the internal root canal morphology may undergo slight modifications over time. In particular, a gradual reduction in canal volume was observed in the *X*-axis groups from 0 to 12 months, whereas the *Y*-axis groups exhibited smaller variations across the same aging intervals. Minor shifts were also detected in centroid; however, these positional changes remained limited in magnitude across all groups. Regarding external morphology, a progressive reduction in total tooth volume was observed with increasing aging time, indicating the occurrence of gradual dimensional contraction of the printed polymer over time. Although the magnitude of this reduction was modest, this trend suggests that post-printing aging may induce slight shrinkage of PolyJet™ 3DPT during storage.

This observation is consistent with the bias analysis reported in the present study, which indicated systematic deviations between 3DPT and the NT. Canal volume and centroid exhibited negative biases, indicating a consistent underestimation of internal structures, whereas total tooth volume and total tooth area showed positive biases, reflecting a systematic overestimation of external features. The range of the 95% LoA increased progressively toward the 12-month evaluation and was consistently greater in the *X*-axis groups. This pattern is consistent with previous studies reporting a gradual reduction in the dimensional stability of 3D-printed specimens over time [15,16,17]. Previous investigations have demonstrated that specimens produced using the PolyJet™ technique in high-quality printing mode may exhibit dimensional expansion, whereby external features tend to be slightly oversized while internal structures are often undersized [14,18]. The present findings are in agreement with this tendency.

PolyJet™ technology is generally associated with low polymerization shrinkage; however, the printing of specimens using pure polymer materials may still result in dimensional changes over time, including shrinkage, warping, and distortion [19]. In the present study, the observed biases and CV indicate that a certain degree of dimensional contraction and distortion occurred within the evaluated time frame, particularly in the *X*-axis groups.

Among all groups, XA0 and YA0 consistently presented the smallest absolute and relative biases and the narrowest 95% LoA across the evaluated variables, indicating closer agreement with the NT. Also, they consistently presented a lower CV compared with the remaining aged groups, indicating that aging may introduce limited measurement dispersion. Nevertheless, visualization of normal deviation through the color deviation maps (Figure 4) demonstrated that most deviations remained within the manufacturer’s stated construction accuracy of approximately 0.1 mm for the Stratasys Objet30 Prime™ printer (Stratasys Ltd., Eden Prairie, MN, USA). Although these deviations are minimal, they should be considered in experimental endodontic studies requiring high morphological precision.

Considering these findings, the differences observed between groups may be explained by printing orientation. When the tooth is printed with the long axis of the root parallel to the *X*-axis, the printer head moves along this direction, resulting in wider deposited layers. In contrast, when the long axis of the root is oriented parallel to the *Y*-axis, the smaller transverse dimension of the root becomes aligned with the *X*-axis movement of the printer head, leading to the deposition of narrower layers. 3D-printed polymers are known to exhibit anisotropic behavior, with mechanical properties dependent on the orientation of the object on the build tray, i.e., the printing direction [2]. This anisotropy is mainly attributed to the layer-by-layer fabrication process, in which adhesion between successive layers is typically weaker than cohesion within each individual layer. Moreover, the orientation of the object in the build platform determines the geometry and number of layers required to fabricate the part and may also influence the exposure of the material to ultraviolet light during curing, potentially affecting the degree of polymerization throughout the structure [2,3,20,21,22]. Accordingly, printing parameters such as layer height, total number of layers, and printing orientation have been identified as key factors influencing distortions and failure mechanisms in PolyJet™ printed specimens, which may occur either between adjacent layers (interlayer failure) or within the same layer (inlayer failure) [19]. In this context, the wider layers generated in the XA groups may increase the structural relevance of interlayer interfaces—recognized as mechanically weaker regions—thereby amplifying anisotropic effects and potentially contributing to the poorer results observed in these specimens [1,21,22]. These findings align with previous studies showing that, even after aging, 3D-printed polymers remain inherently anisotropic, with mechanical properties governed by the printing direction [2]. Although in our previous study [6] the build position appeared to be more relevant than the printing axis, since no significant differences were found between the X and Y groups at 0 months, the present results suggest that aging makes the effect of printing axis more evident, likely due to the combined influence of anisotropy, weaker interlayer interfaces, and orientation-dependent curing effects.

Previous studies have reported that the PolyJet™ glossy finishing, which leaves the topmost specimen surfaces uncovered by support material, may produce superior overall printing results [17]; nonetheless, finishing operations (glossy versus matte) have been shown not to significantly affect the tensile strength of 3D-printed specimens, although they may influence surface roughness properties [4]. Also, it is stated that material selection has a significant influence on the properties of glossy and matte finishing [19]. In the present study, a matte finishing was selected because 3DPT require homogeneous surface characteristics. In this mode, the printed specimen is completely embedded in support material, including the topmost surfaces, ensuring surface evenness. For complex anatomical geometries, achieving uniform glossy surfaces is often not feasible; therefore, matte finishing represents the most suitable option [4,19]. Additionally, matte finishing enables less dimensional deviation and the reproduction of sharper edges, as the specimens are fully supported by the printing material, which is essential for accurately replicating fine anatomical details [4].

However, an unresolved issue concerns whether, and to what extent, moisture from the support material may influence the properties of printed specimens. Previous studies have shown that humidity may significantly affect the dimensional and mechanical properties of PolyJet™ printed polymers, particularly during prolonged aging [11,22], since moisture uptake by polymer-based materials can lead to significant alterations in their material properties [23]. Since support material may act as a partial barrier to moisture absorption [9], the 3DPT in the present study were stored with the support material in place in order to minimize environmental humidity effects and better isolate the influence of aging on the evaluated parameters. In addition, although the support removal protocol described by Reis et al. (2024) [14] uses alcohol as the last irrigant after water irrigation, given its proposed role in removing residual moisture from the root canal in endodontics [24], the protocol inherently involves exposure to water, which may influence mechanical behavior over time through moisture absorption or evaporation, potentially affecting material properties [1,4]. Therefore, the support material was retained during storage also to minimize protocol-induced moisture effects and ensure controlled aging conditions.

Another issue is the lightning conditions. In the literature, there exists reports showing that no significant changes in material properties occurred under different lighting conditions [22], even when specimens were exposed to potential additional ultraviolet radiation from everyday lighting. However, polymer degradation during artificial aging is known to be associated with ultraviolet exposure. Ultraviolet radiation can induce alterations in the polymer molecular chains, including cross-linking and reductions in molecular weight, which may be detected through changes in mechanical properties and color [21]. Considering this potential influence of light exposure, the 3DPT in the present study were stored in a closed opaque plastic container to further minimize uncontrolled light exposure during the aging period.

Regarding the secondary aim, the present findings do not support the null hypothesis that aging does not affect the shaping behavior of 3DPT during endodontic preparation. The micro-CT based analysis demonstrated that aging influenced key endodontic preparation-related parameters, including percentage increase in canal volume, percentage of unprepared areas, and centroid deviation, indicating that the material response to endodontic preparation is altered over time.

The distinct patterns observed between printing orientations further support this interpretation. In the *X*-axis groups, the progressive differences suggest a cumulative effect of aging, whereas in the *Y*-axis groups, the absence of differences between intermediate and longer aging intervals indicates that most changes occur early, with limited additional variation over time. These findings agree with previous reports indicating that aging-related modifications tend to stabilize after the initial post-fabrication period. Notably, the most pronounced alterations in mechanical properties have been reported during the initial aging period, followed by a phase of relative stabilization, which aligns with the plateau observed in the *Y*-axis groups in the present study [1,4,22,25].

Although significant differences were identified in centroid deviation, canal transportation remained unchanged across aging conditions. This suggests that aging may influence the direction of canal deviation without affecting its overall magnitude, indicating that localized variations in material response do not necessarily translate into changes in the global endodontic preparation.

The observed differences in the percentage increase in canal volume and unprepared areas further suggest that aging influences the interaction between the endodontic instrument and canal walls. The previous literature reported time-dependent changes in the mechanical behavior of PolyJet™ materials [4,21,22,25]. Aging has been associated with increased ultimate tensile strength and reduced elongation, while the elastic modulus tends to remain relatively stable. This combination suggests a progressive shift toward more brittle behavior, despite reports that some rigid materials may also become more deformable over time [2,4,8,25]. Such changes are commonly attributed to post-curing processes and physicochemical stabilization occurring after fabrication [1,8,21,22]. In this way, material behavior may alter cutting dynamics, potentially leading to less uniform endodontic instrument engagement and more heterogeneous endodontic preparation outcomes. 

Overall, these findings indicate that aging induces measurable changes in the shaping behavior of PolyJet™ 3DPT, which are consistent with previously described time-dependent modifications in polymer-based materials. These results reinforce the importance of considering storage time and printing orientation when designing experimental studies, as both factors may significantly influence endodontic preparation behavior and the reproducibility of outcomes.

One of the principal objectives underlying the development and use of 3DPT is the achievement of greater standardization in both experimental and educational settings. When NT are used, the establishment of well-balanced experimental groups in ex vivo studies, as well as the implementation of fair and comparable student assessments in preclinical training, is inherently challenging due to the complex and highly variable anatomy of the root canal system. Consequently, differences observed between experimental groups may reflect anatomical variability rather than the true effect of the variable under investigation [26]. In educational contexts, this variability also represents a recognized limitation when NT are used as training models. Students frequently report that variations in canal morphology compromise the fairness and validity of individual performance assessments, as the degree of procedural difficulty may vary considerably between NT [27].

Furthermore, intrinsic biological variability in dentin further complicates attempts to achieve true standardization. Dentin is a complex hierarchical tissue composed of approximately 45% mineral, 33% organic matrix, and 22% water, characterized by a tubular microstructure whose density and degree of mineralization change with age due to progressive tubule occlusion. These structural changes result in alterations in mechanical properties and contribute to significant variability between teeth and individuals. For these reasons, complete standardization when using NT is, with current methodologies, extremely difficult to achieve [3].

In this context, 3DPT represent a promising alternative for reducing biological variability and improving experimental and educational reproducibility. However, the results of the present study and of our previous study [6] indicate that achieving the intended level of standardization also requires careful control of manufacturing conditions. For experimental purposes, it is recommended that all specimens be produced within the same printing batch and, whenever possible, used shortly after fabrication to minimize potential dimensional changes associated with storage or material aging. Similarly, in educational settings, distributing 3DPT originating from the same printing batch to all students may enhance standardization and improve the consistency of training conditions. Nevertheless, in university environments, cost-effectiveness and logistical constraints must be considered, and the use of freshly printed specimens may not always be feasible. In such cases, printing orientation becomes a relevant factor. Based on the present findings, specimens printed along the *Y*-axis demonstrated greater stability over time, suggesting that this orientation may be preferable when storage prior to use is unavoidable. Adopting this strategy may help mitigate aging-related effects while maintaining an acceptable level of standardization in preclinical training.

The present study shares several limitations with our previous study [6]. First, the analysis was restricted to a single tooth presenting a specific anatomical configuration, and all specimens were produced using a single PolyJet™ printer. As a result, the findings may not fully represent the variability that may arise from different anatomical morphologies or printing systems. Furthermore, multiple factors intrinsic to PolyJet™ technology can influence the final geometry of the printed specimens. In addition, only one photopolymer resin and a single type of support material were employed, which may limit the generalizability of the results to other material combinations. Despite these limitations, the findings highlight important considerations for both research and educational applications involving 3DPT. Future investigations should also evaluate a broader range of anatomical configurations, including more complex canal morphologies such as lateral or recurrent canals. Exploring these variations would allow a more comprehensive assessment of how anatomical complexity ages and affects the dimensional accuracy of PolyJet™ 3DPT. It has also been reported that reinforcing materials can be incorporated into the polymer matrix to enhance mechanical strength and mitigate printing-related limitations associated with the PolyJet™ technique [19]. In this context, the use of reinforced materials may also help overcome some of the limitations reported for 3DPT in the literature, particularly the reduced radiopacity and hardness compared with natural dental tissues [28]; however, further research is required to determine whether these materials can effectively address these shortcomings.

It is widely recognized that improving the quality of additively manufactured models depends on a detailed understanding of the interaction between material properties, processing parameters, and the resulting structural characteristics. Such knowledge is essential for maximizing the potential of 3D printing technologies, and manufacturing imperfections should be minimized through optimization strategies that account for dimensional deviations and systematically evaluate the design [9,19]. Considering the present findings, future studies involving PolyJet™ 3DPT should clearly report whether the specimens originated from the same printing batch and specify the storage time between fabrication and experimental use. The consistent reporting of these parameters will facilitate comparisons between studies and contribute to a more reliable interpretation of results, particularly with regard to their potential translation to clinical scenarios.

## 5. Conclusions

Within the limitations of the present study, aging had a limited effect on the dimensional accuracy of PolyJet™ 3DPT, with only small deviations that did not compromise overall geometry. However, specimens printed with tooth orientation along the *X*-axis showed greater aging-related deviations than those printed along the *Y*-axis. In contrast, aged 3DPT demonstrated measurable differences in canal shaping outcomes during root canal preparation with PTG, namely in canal volume increase, unprepared areas, and centroid deviation, indicating that aging can substantially influence endodontic preparation outcomes. Based on these findings, future studies using PolyJet™ 3DPT should clearly report whether the specimens originate from the same printing batch and specify the storage time between fabrication and experimental use. The consistent reporting of these parameters will facilitate comparisons between studies and support a more reliable interpretation of results, particularly regarding their potential relevance to clinical scenarios.

## Figures and Tables

**Table 1:** Micro-computed tomographic analysis before endodontic preparation of natural tooth and 3D-printed teeth.

	Natural Tooth		3D-Printed Teeth					
			XA0	XA6	XA12	YA0	YA6	YA12
Canal Volume (mm^3^)	12.40	Mean ± SD	10.23 ± 0.31	9.76 ± 0.82	9.39 ± 0.43	10.58 ± 0.31	10.51 ± 0.41	10.17 ± 0.48
		Median	10.28	9.83	9.47	10.54	10.46	10.13
		Min–Max	9.72–10.67	8.10–11.20	8.70–9.86	10.19–11.19	10.06–11.27	9.37–10.96
Centroid X (mm)	13.28	Mean ± SD	13.20 ± 0.03	13.19 ± 0.04	13.19 ± 0.04	13.16 ± 0.05	13.15 ± 0.03	13.16 ± 0.03
		Median	13.20	13.18	13.20	13.17	13.16	13.16
		Min–Max	13.15–13.25	13.15–13.27	13.14–13.26	13.02–13.22	13.11–13.19	13.12–13.21
Centroid Y (mm)	12.64	Mean ± SD	12.52 ± 0.04	12.51 ± 0.03	12.51 ± 0.03	12.54 ± 0.02	12.54 ± 0.02	12.54 ± 0.04
		Median	12.53	12.51	12.50	12.54	12.55	12.54
		Min–Max	12.45–12.58	12.46–12.57	12.46–12.58	12.49–12.57	12.51–12.56	12.49–12.60
Centroid Z (mm)	11.20	Mean ± SD	11.13 ± 0.06	11.14 ± 0.11	11.21 ± 0.05	11.10 ± 0.05	11.13 ± 0.02	11.14 ± 0.04
		Median	11.12	11.13	11.21	11.10	11.14	11.15
		Min–Max	11.07–11.25	11.01–11.32	11.14–11.27	11.02–11.20	11.09–11.16	11.08–11.17
Total Tooth Volume (mm^3^)	381.45	Mean ± SD	387.56 ± 3.23	382.56 ± 6.92	379.91 ± 5.56	389.27 ± 3.19	388.53 ± 3.33	382.98 ± 4.04
		Median	387.40	381.86	379.27	389.87	388.30	384.26
		Min–Max	381.75–392.30	371.55–393.36	371.56–389.69	384–393.54	384.34–395.03	372.93–387.43
Total Tooth Area (mm^2^)	430.00	Mean ± SD	454.24 ± 2.85	457.63 ± 7.91	453.28 ± 6.99	450.72 ± 2.65	450.44 ± 1.85	445.21 ± 3.41
		Median	454.16	456.14	451.31	451.11	450.71	445.80
		Min–Max	450.60–460.84	450.74–471.67	445.40–467.15	445.82–454.30	447.04–453.91	437.49–449.63

**Table 2:** Absolute (mm, mm^2^, mm^3^) and relative (%) biases of 3D-printed teeth groups compared with the natural tooth and corresponding 95% limits of agreement (95% LoA).

		3D Printed Teeth					
		XA0	XA6	XA12	YA0	YA6	YA12
Canal Volume (mm^3^)	Bias	−2.17	−2.64	−3.00	−1.83	−1.89	−2.23
	95% LoA	−2.78 to −1.56	−4.24 to −1.03	−3.84 to −2.16	−2.44 to −1.22	−2.69 to −1.09	−3.16 to −1.29
Centroid X (mm)	Bias	−0.08	−0.09	−0.09	−0.12	−0.13	−0.12
	95% LoA	−0.14 to −0.02	−0.16 to −0.02	−0.18 to −0.00	−0.22 to −0.02	−0.18 to −0.08	−0.17 to −0.07
Centroid Y (mm)	Bias	−0.12	−0.13	−0.13	−0.10	−0.10	−0.10
	95% LoA	−0.20 to −0.04	−0.19 to −0.07	−0.20 to −0.06	−0.14 to −0.06	−0.13 to −0.07	−0.18 to −0.02
Centroid Z (mm)	Bias	−0.07	−0.06	0.01	−0.10	−0.07	−0.06
	95% LoA	−0.19 to 0.05	−0.27 to 0.15	−0.09 to 0.10	−0.20 to 0.00	−0.11 to −0.02	−0.11 to 0.01
Total Tooth Volume (mm^3^)	Bias	6.11	1.11	−1.54	7.82	7.08	1.53
	95% LoA	−0.22 to 12.44	−12.48 to 14.70	−12.45 to 9.36	1.57 to 14.07	1.24 to 12.91	−6.40 to 9.45
Total Tooth Area (mm^2^)	Bias	24.24	27.63	23.28	20.72	20.44	15.21
	95% LoA	18.65 to 29.83	12.12 to 43.13	9.58 to 36.99	15.53 to 25.91	16.80 to 24.07	8.52 to 21.90
Canal Volume (%)	Bias	−17.52	−21.29	−24.20	−14.78	−15.24	−17.98
	95% LoA	−22.48 to −12.56	−34.19 to −8.39	−30.95 to −17.45	−19.72 to −9.84	−21.68 to −8.80	−25.48 to −10.48
Centroid X (%)	Bias	−0.59	−0.67	−0.66	−0.90	−0.96	−0.90
	95% LoA	−1.10 to −0.08	−1.21 to 0.13	−1.32 to −0.01	−1.70 to −0.10	−1.33 to −0.59	−1.32 to −0.49
Centroid Y (%)	Bias	−0.94	−1.04	−1.04	−0.81	−0.78	−0.78
	95% LoA	−1.51 to −0.37	−1.51 to −0.56	−1.58 to −0.50	−1.16 to −0.46	−1.01 to −0.54	−1.38 to −0.18
Centroid Z (%)	Bias	−0.60	−0.54	0.05	−0.88	−0.62	−0.54
	95% LoA	−1.58 to 0.38	−2.43 to 1.36	−0.81 to 0.92	−1.74 to −0.02	−1.02 to −0.22	−1.16 to −0.09
Total Tooth Volume (%)	Bias	1.60	0.29	−0.40	2.05	1.86	0.40
	95% LoA	−0.07 to −3.27	−3.27 to 3.85	−3.26 to 2.45	0.40 to 3.70	0.33 to 3.39	−1.68 to 2.48
Total Tooth Area (%)	Bias	5.64	6.43	5.41	4.82	4.75	3.54
	95% LoA	4.35 to 6.93	2.82 to 10.04	2.23 to 8.59	3.60 to 6.04	3.91 to 5.59	1.99 to 5.09

**Table 3:** Coefficient of variation in total tooth volume and total tooth area for each 3D-printed teeth group.

		3D-Printed Teeth					
		XA0	XA6	XA12	YA0	YA6	YA12
Total Tooth Volume (mm^3^)	Mean ± SD	387.56 ± 3.23	382.56 ± 6.93	379.91 ± 5.56	389.27 ± 3.19	389.53 ± 3.33	382.97 ± 4.04
	Coefficient of Variation	0.83	1.81	1.46	0.82	0.83	1.05
Total Tooth Area (mm^2^)	Mean ± SD	454.24 ± 2.85	457.62 ± 7.91	453.28 ± 6.99	450.72 ± 2.65	450.44 ± 1.85	445.21 ± 3.41
	Coefficient of Variation	0.63	1.73	1.54	0.59	0.41	0.76

**Table 4:** Micro-computed tomographic analysis of groups XA and YA before and after preparation with ProTaper Gold^®^.

			XA0	XA6	XA12	YA0	YA6	YA12
Canal Volume (mm^3^)	Initial	Mean ± SD	10.23 ± 0.31	9.76 ± 0.82	9.39 ± 0.43	10.58 ± 0.31	10.51 ± 0.41	10.17 ± 0.48
		Min–Max	9.72–10.67	8.10–11.20	8.70–9.86	10.19–11.19	10.06–11.27	9.37–10.96
	After	Mean ± SD	17.97 ± 0.52	18.84 ± 0.63	16.64 ± 1.02	18.25 ± 0.50	19.15 ± 0.55	16.37 ± 0.56
		Min–Max	17.20–18.73	17.90–19.60	15.20–17.86	17.21–18.83	18.10–19.81	15.68–17.42
	% Volume Increase	Mean ± SD	75.87 ± 7.20	93.88 ± 12.45	77.09 ± 8.54	72.87 ± 7.72	82.44 ± 7.61	61.12 ± 7.12
		Min–Max	63.54–86.34	75.00–121.73	59.33–85.46	53.80–81.94	70.19–94.69	43.52–70.65
Centroid X (mm)	Initial	Mean ± SD	13.20 ± 0.03	13.19 ± 0.04	13.19 ± 0.04	13.16 ± 0.05	13.15 ± 0.03	13.16 ± 0.03
		Min–Max	13.15–13.25	13.15–13.27	13.14–13.26	13.02–13.22	13.11–13.19	13.12–13.21
	After	Mean ± SD	13.66 ± 0.04	13.56 ± 0.04	13.65 ± 0.06	13.64 ± 0.03	13.53 ± 0.02	13.54 ± 0.04
		Min–Max	13.58–13.72	13.52–13.65	13.56–13.74	13.59–13.70	13.49–13.56	13.46–13.58
	Deviation	Mean ± SD	0.46 ± 0.06	0.37 ± 0.02	0.46 ± 0.06	0.48 ± 0.06	0.37 ± 0.03	0.38 ± 0.05
		Min–Max	0.39–0.54	0.34–0.40	0.41–0.58	0.41–0.63	0.33–0.42	0.30–0.44
Centroid Y (mm)	Initial	Mean ± SD	12.52 ± 0.04	12.51 ± 0.03	12.51 ± 0.03	12.54 ± 0.02	12.54 ± 0.02	12.54 ± 0.04
		Min–Max	12.45–12.58	12.46–12.57	12.46–12.58	12.49–12.57	12.51–12.56	12.49–12.60
	After	Mean ± SD	12.99 ± 0.03	12.88 ± 0.04	12.95 ± 0.05	13.02 ± 0.02	12.92 ± 0.06	12.96 ± 0.04
		Min–Max	12.92–13.02	12.80–12.94	12.86–13.02	12.98–13.07	12.82–12.99	12.89–13.01
	Deviation	Mean ± SD	0.46 ± 0.06	0.37 ± 0.03	0.45 ± 0.03	0.49 ± 0.03	0.38 ± 0.05	0.42 ± 0.03
		Min–Max	0.34–0.55	0.30–0.42	0.40–0.50	0.42–0.54	0.31–0.45	0.37–0.46
Centroid Z (mm)	Initial	Mean ± SD	11.13 ± 0.06	11.14 ± 0.11	11.21 ± 0.05	11.10 ± 0.05	11.13 ± 0.02	11.14 ± 0.04
		Min–Max	11.07–11.25	11.01–11.32	11.14–11.27	11.02–11.20	11.09–11.16	11.08–11.17
	After	Mean ± SD	11.44 ± 0.04	11.56 ± 0.04	11.41 ± 0.11	11.45 ± 0.04	11.43 ± 0.02	11.42 ± 0.05
		Min–Max	11.35–11.48	11.51–11.63	11.13–11.56	11.38–11.53	11.38–11.46	11.32–11.49
	Deviation	Mean ± SD	0.30 ± 0.08	0.42 ± 0.08	0.21 ± 0.14	0.35 ± 0.08	0.30 ± 0.03	0.28 ± 0.06
		Min–Max	0.20–0.41	0.28–0.53	−0.11–0.42	0.23–0.45	0.25–0.37	0.18–0.37
Canal Transportation (mm)		Mean ± SD	0.72 ± 0.08	0.77 ± 0.06	0.68 ± 0.08	0.77 ± 0.06	0.61 ± 0.06	0.64 ± 0.07
		Min–Max	0.57–0.84	0.66–0.86	0.60–0.87	0.66–0.86	0.53–0.71	0.51–0.72
Unprepared area (%)		Mean ± SD	36.93 ± 2.99	27.68 ± 3.89	35.08 ± 6.18	38.18 ± 3.82	32.84 ± 4.52	40.16 ± 2.75
		Min–Max	32.89–42.24	22.71–33.77	23.33–45.34	31.96–44.58	25.33–37.21	36.86–46.27

## Data Availability

The data that supports the findings of this study are available from the corresponding author upon reasonable request. The data is not publicly available due to privacy and ethical restrictions (undergoing PhD thesis).

## Abbreviations

The following abbreviations are used in this manuscript:

3D	Three-dimensional
3DPT	Three-dimensional printed teeth
Micro-CT	Micro–computed tomography
NT	Natural Teeth
PTG	ProTaper Gold^®^
SD	Standard deviation
LoA	Limit of agreement
CV	Coefficient of variation
